# Exploring Healthy Retinal Aging with Deep Learning

**DOI:** 10.1016/j.xops.2023.100294

**Published:** 2023-03-01

**Authors:** Martin J. Menten, Robbie Holland, Oliver Leingang, Hrvoje Bogunović, Ahmed M. Hagag, Rebecca Kaye, Sophie Riedl, Ghislaine L. Traber, Osama N. Hassan, Nick Pawlowski, Ben Glocker, Lars G. Fritsche, Hendrik P.N. Scholl, Sobha Sivaprasad, Ursula Schmidt-Erfurth, Daniel Rueckert, Andrew J. Lotery

**Affiliations:** 1 BioMedIA, Imperial College London, London, United Kingdom; 2 Institute for AI and Informatics in Medicine, Technical University of Munich, Munich, Germany; 3 Laboratory for Ophthalmic Image Analysis, Medical University of Vienna, Vienna, Austria; 4 Christian Doppler Laboratory for Artificial Intelligence in Retina, Christian Doppler Forschungsgesellschaft, Vienna, Austria; 5 Institute of Ophthalmology, University College London, London, United Kingdom; 6 Moorfields Eye Unit, National Institute for Health Research, London, United Kingdom; 7 Clinical and Experimental Sciences, Faculty of Medicine, University of Southampton, Southampton, United Kingdom; 8 Institute of Molecular and Clinical Ophthalmology Basel, Basel, Switzerland; 9 Department of Ophthalmology, University of Basel, Basel, Switzerland; 10 Microsoft Research, Cambridge, United Kingdom; 11 Department of Biostatistics, University of Michigan, Ann Arbor, Michigan

**Keywords:** Aging, Biomarker discovery, Deep learning, Machine learning, Retina

## Abstract

**Purpose:**

To study the individual course of retinal changes caused by healthy aging using deep learning.

**Design:**

Retrospective analysis of a large data set of retinal OCT images.

**Participants:**

A total of 85 709 adults between the age of 40 and 75 years of whom OCT images were acquired in the scope of the UK Biobank population study.

**Methods:**

We created a counterfactual generative adversarial network (GAN), a type of neural network that learns from cross-sectional, retrospective data. It then synthesizes high-resolution counterfactual OCT images and longitudinal time series. These counterfactuals allow visualization and analysis of hypothetical scenarios in which certain characteristics of the imaged subject, such as age or sex, are altered, whereas other attributes, crucially the subject’s identity and image acquisition settings, remain fixed.

**Main Outcome Measures:**

Using our counterfactual GAN, we investigated subject-specific changes in the retinal layer structure as a function of age and sex. In particular, we measured changes in the retinal nerve fiber layer (RNFL), combined ganglion cell layer plus inner plexiform layer (GCIPL), inner nuclear layer to the inner boundary of the retinal pigment epithelium (INL-RPE), and retinal pigment epithelium (RPE).

**Results:**

Our counterfactual GAN is able to smoothly visualize the individual course of retinal aging. Across all counterfactual images, the RNFL, GCIPL, INL-RPE, and RPE changed by −0.1 μm ± 0.1 μm, −0.5 μm ± 0.2 μm, −0.2 μm ± 0.1 μm, and 0.1 μm ± 0.1 μm, respectively, per decade of age. These results agree well with previous studies based on the same cohort from the UK Biobank population study. Beyond population-wide average measures, our counterfactual GAN allows us to explore whether the retinal layers of a given eye will increase in thickness, decrease in thickness, or stagnate as a subject ages.

**Conclusion:**

This study demonstrates how counterfactual GANs can aid research into retinal aging by generating high-resolution, high-fidelity OCT images, and longitudinal time series. Ultimately, we envision that they will enable clinical experts to derive and explore hypotheses for potential imaging biomarkers for healthy and pathologic aging that can be refined and tested in prospective clinical trials.

**Financial Disclosure(s):**

Proprietary or commercial disclosure may be found after the references.

Many retinal diseases, such as age-related macular degeneration and diabetic retinopathy, develop gradually over time.^1^^,^^2^ Clinicians are able to track their progression using OCT imaging, which provides high-resolution images of the retina.^3^ However, the retina also undergoes age-related physiologic changes.^4^ A good understanding of how healthy aging manifests itself in the retina is a crucial prerequisite to distinguish between normal and pathologic changes and effectively diagnose, prognose, and treat ocular diseases.

The retina has been extensively studied by retrospectively or prospectively collecting large amounts of OCT images from representative populations.^5^, ^6^, ^7^, ^8^, ^9^, ^10^, ^11^, ^12^, ^13^, ^14^ The pooled images are analyzed by measuring the shape and thickness of individual retinal layers. By identifying population-wide correlations between the eyes’ structure and demographic, lifestyle, and medical information, researchers are able to find and validate imaging biomarkers. Supported by the emergence of large population studies and automated tools for processing of medical images,^15^ these approaches have successfully found links between age and changes in the nerve fiber layer,^5^^,^^6^^,^^8^^,^^14^ ganglion cell complex,^7^, ^8^, ^9^^,^^14^ photoreceptor layers,^8^^,^^13^ and retinal pigment epithelium (RPE).^12^

However, these population-based studies have several shortcomings. Usually, pooled data sets only include a single scan of each eye. Even if time series data are available, it is rare that a subject is monitored for longer than a couple of years. Furthermore, the imaging conditions change between subsequent visits. The retina may appear differently because of varying levels of pupil dilation, changes in OCT scanner hardware and software, and different orientations of the eye. Consequently, population-based studies are limited in their ability to evaluate the development of the eye at a subject-specific level and resolve subtle retinal changes that occur over the course of decades.

In this study, we used deep learning to study the individual course of retinal changes caused by healthy aging. Our counterfactual generative adversarial network (GAN), a type of neural network, learns from cross-sectional retrospective data. It then synthesizes high-resolution counterfactual OCT images and longitudinal time series. These counterfactuals reflect hypothetical scenarios in which certain characteristics of the imaged subject, such as age or sex, are altered, whereas other attributes, crucially the subject’s identity and image acquisition settings, remain fixed. Such counterfactual images allow the investigation of what-if questions that are impossible to answer in population-based studies. Examples of such counterfactual queries are “how will this person’s eye look in 20 years?” or “how would this eye look if the subject was born as the opposite sex?” We extensively benchmark the visual fidelity and realism of the generated counterfactual images before ultimately demonstrating the utility of our proposed method by quantifying the subject-specific retinal layer structure as a function of age and sex.

## Methods

**Figure 1:**
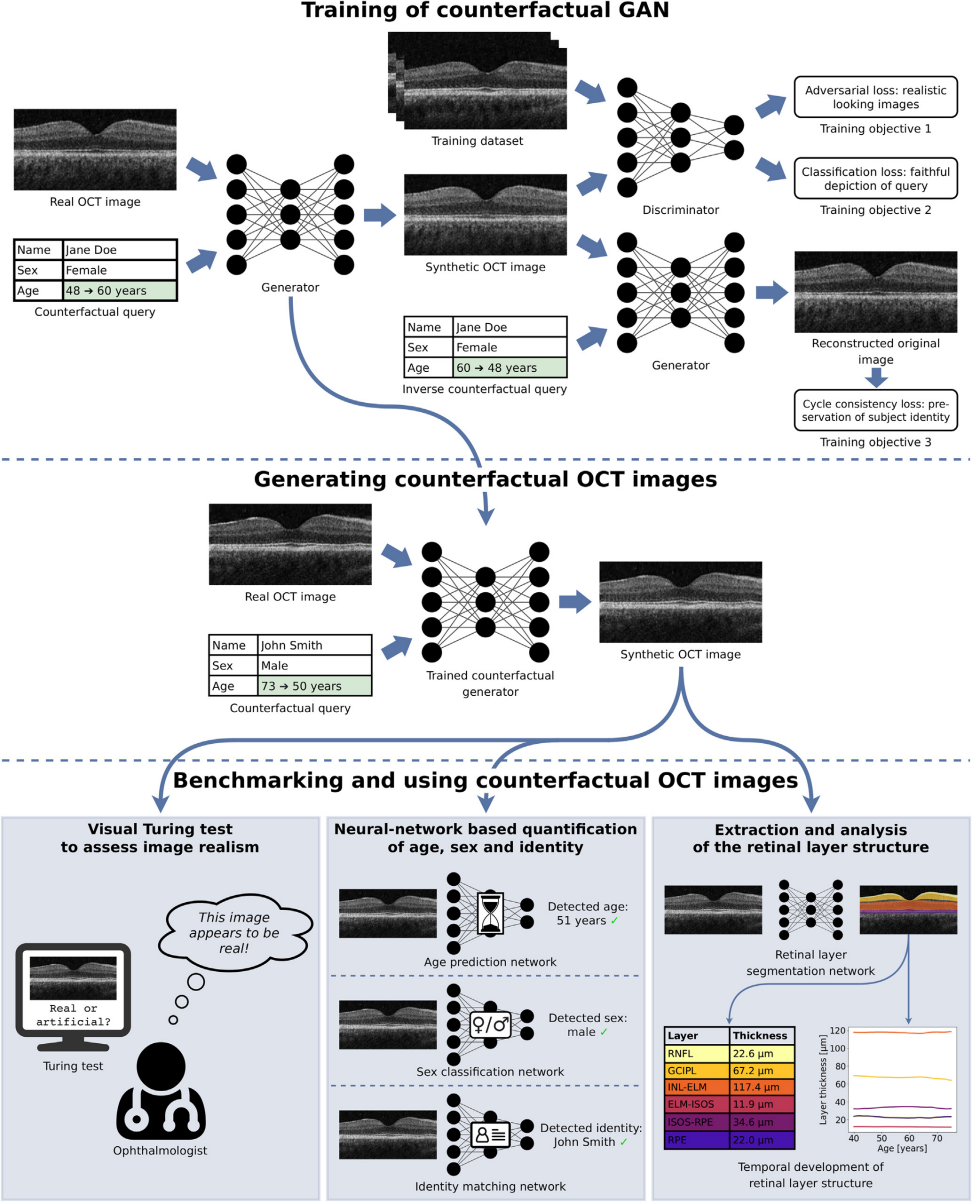
Workflow diagram explaining how the counterfactual generative adversarial network (GAN) is trained (top part), used to generate counterfactual OCT images (middle part) and benchmarked and utilized (bottom part). ELM = external limiting membrane; GCIPL = ganglion cell layer plus inner plexiform layer; INL = inner nuclear layer; ISOS = inner segment/outer segment junction layer; RNFL = retinal nerve fiber layer; RPE = retinal pigment epithelium.

An overview of our method and study workflow is presented in Figure 1. After introducing the used data set of OCT images, we describe the counterfactual GAN. Next, we present our experiments to measure the visual fidelity and realism of the artificial OCT images, respectively. Finally, we describe how to extract and analyze the retinal layer structure from the counterfactual images.

### Participants and OCT Image Data Set

We used the OCT image data set that has been acquired as part of the UK Biobank population study. The UK Biobank has collected extensive demographic, lifestyle, health, and medical imaging information from > 500 000 members of the United Kingdom’s general public.^16^ In its scope, 175 844 retinal OCT scans of 85 709 participants were acquired using a Topcon 3D OCT-1000 Mark II device (Topcon Corporation).^17^^,^^18^ The UK Biobank population study has been reviewed and approved by the North West Multicentre Research Ethics Committee in accordance with the tenets of the Declaration of Helsinki so that additional ethical approval was not required for our study.

**Figure 2:**
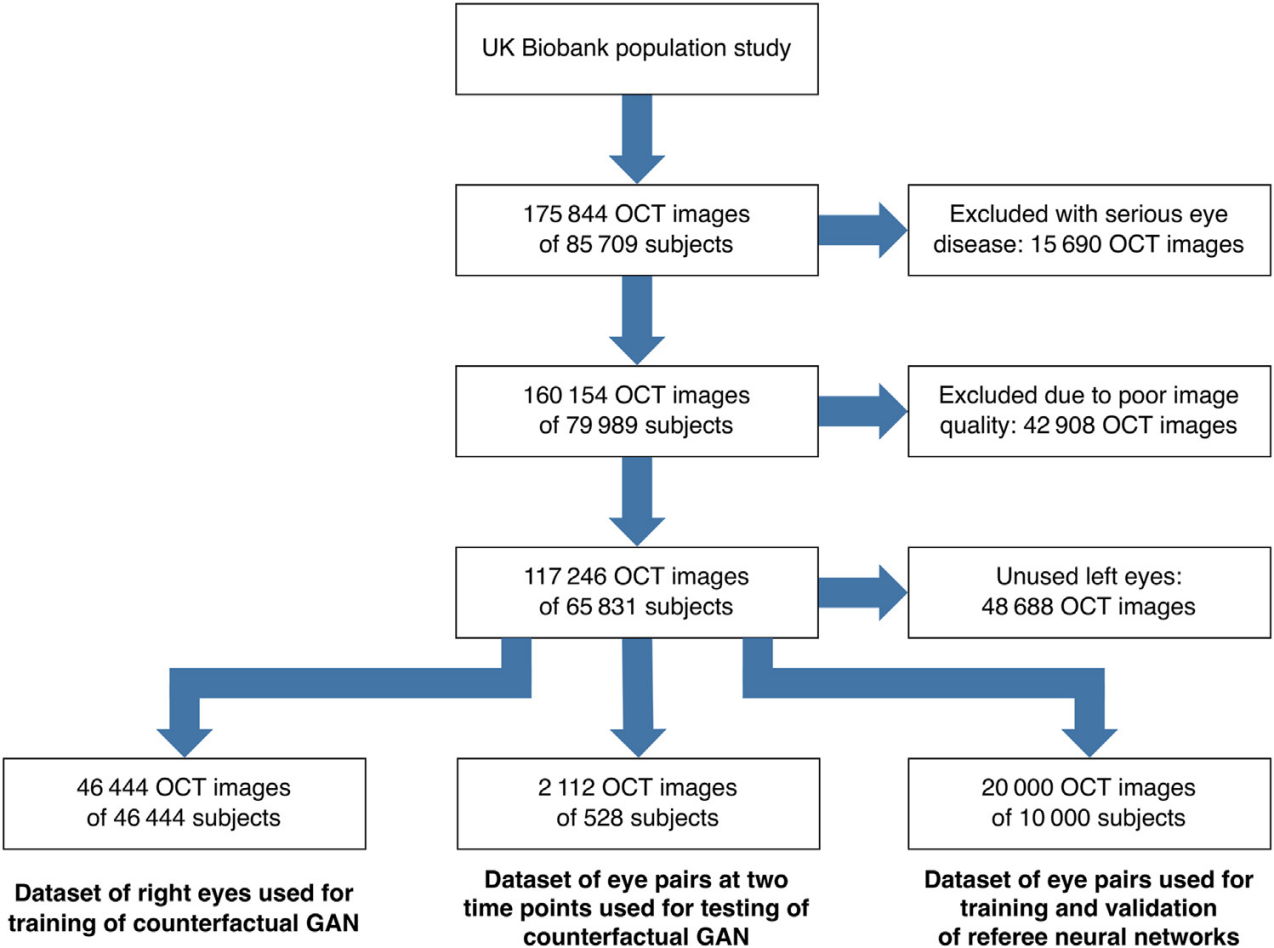
Data flowchart presenting the data inclusion and exclusion criteria as well as the final split into the 3 independent data sets used in the study. GAN = generative adversarial network.

During image preprocessing, we filtered out scans of poor image quality using an intensity-histogram–based score (Fig 2).^19^ We also excluded any subjects that reported being affected by age-related macular degeneration, diabetic retinopathy, glaucoma, cataracts, previous eye trauma, or other serious eye diseases. Next, 11 retinal layer surfaces of the 3-dimensional OCT scans were segmented using the Iowa Reference Algorithms (Retinal Image Analysis Laboratory, Iowa Institute for Biomedical Imaging).^20^, ^21^, ^22^ The obtained layer segmentations were used to flatten and register all images. During flattening, the images were sheared so that the outer boundary of the RPE is orientated horizontally. The center of the fovea was defined as the position with the minimal distance between the inner limiting membrane and the outer plexiform layer. We extracted the transverse 2-dimensional slice that passed through this position. Finally, all images were resampled to 224 × 224 pixels with a pixel size of 23.4 × 7.0 μm^2^, half the median resolution.

Preprocessing yielded 117 246 highly standardized images of 65 831 subjects. The data set was then split into 3 subdata sets (Fig 2). Overall, 46 444 scans of the right eye of 46 444 subjects were used for training of the counterfactual GAN. A total of 20 000 images of 10 000 eye pairs were used to train a set of referee neural networks that evaluated the generated images. Overall, 2112 images of 528 subjects, for which initial and follow-up scans of both eyes were available, were used for final testing. Because images of left eyes were only used for training of the referee networks and final testing but not for GAN training, we ended up not using 48 688 images from our data set.

### GAN to Synthesize Counterfactual OCT Images

The task of generating counterfactual OCT images was formulated as image translation using a GAN.^23^ Our counterfactual GAN consists of 2 neural networks, a generator, and a discriminator (Fig 1). The generator is provided with a real OCT image and a counterfactual query, which consists of the target age and sex encoded as a vector. Given these inputs, the generator is tasked with creating a counterfactual image. These images are provided to the discriminator together with a set of real images from the training data set. For each image, the discriminator has to establish whether it is real or artificially generated. The discriminator also has to estimate the age and sex of the subject in each case. Finally, the counterfactual images are passed through the generator once again with the goal of changing their appearance back to their original state.

Based on these training objectives, the generator and discriminator are trained simultaneously in a zero-sum game.

During inference, the trained generator receives an existing real OCT image and a counterfactual query and creates a corresponding counterfactual image (Fig 1). The neural network framework was adapted from work by Choi et al.^24^ We describe our modifications to it and the full network architecture, training procedure, and used hyperparameters in the supplemental material (available at www.ophthalmologyscience.org).

### Visual Turing Test to Assess Image Realism

To quantitatively assess the realism of the counterfactual images, we conducted a visual Turing test. The test measured the ability of 5 expert ophthalmologists (A.M.H., R.K., S.R., G.L.T., A.J.L.) to distinguish between real and artificially generated OCT images. To ensure a fair comparison, all real images were downsampled and flattened according to the previously reported image preprocessing steps. Initially, the participants were given the option to review up to 100 real images. Afterward, they were shown 50 real OCT images and 50 artificially generated images in random order and had to determine which ones were real and which ones were fake. We report the average accuracy of all ophthalmologists.

### Neural-Network–Based Quantification of Counterfactual Age, Sex, and Identity

It is crucial that the counterfactual GAN faithfully models the effect of subject age and sex, whereas simultaneously preserving the subject identity. To measure this capability, we trained 3 referee neural networks built according to the well-established Resnet50 architecture.^25^ The age prediction network estimates the subject’s age from an input OCT image. The sex classification network predicts whether a given OCT image belongs to a male or female subject. The identity-matching network learns to assign a similarity score to an image pair consisting of right and left eyes. A high similarity score indicates that the 2 eyes belong to the same subject. Full network configuration and training procedure are included in the supplemental material.

Before ultimately using the 3 referee networks to evaluate the counterfactual images, we benchmarked their performance on an independent subset of real OCT images. The age regression network estimated the subject’s age with a mean absolute error of 4.1 years. The sex classification network determined the subject’s sex with an accuracy of 79.5% and an area under the receiver operating characteristic curve of 0.90. The identity-matching network was tasked with matching 2000 right and left eyes belonging to 1000 different subjects and achieved a sensitivity of 95.8% and a specificity of 97.7%. During evaluation, the 3 referee networks were shown 10 000 counterfactual images. The counterfactual queries were evenly split between male and female sex and distributed across a uniform age distribution spanning from 40 to 75 years, the age range of the subjects in the training data set. We report whether the determined age, sex, and identity matched the corresponding counterfactual queries.

### Extraction and Analysis of the Retinal Layer Structure

Finally, we analyzed the retinal structure in the OCT images. To this end, we trained a Resnet50 neural network to segment 11 retinal surfaces following the approach by Shah et al.^26^ We used real OCT images and the previously obtained layer segmentations as training data. The network was able to accurately localize the retinal layers in an independent subset of 1000 real OCT images. Across all 11 layers, the mean absolute difference between the predicted and ground truth layer segmentations was 4.2 μm ± 7.5 μm. Because we did not have ground truth annotations for the counterfactual OCT images, we could not quantitatively assess the segmentation network’s performance on these images. However, we visually confirmed that the network is able to delineate the layers in artificially generated images before proceeding to process all counterfactual images. Additionally, we segmented and analyzed the real OCT images that were used to train the counterfactual GAN, resembling conventional population-based studies.^12^, ^13^, ^14^ More details can be found in the supplementary material.

In this study, we focused on the retinal nerve fiber layer (RNFL), combined ganglion cell layer plus inner plexiform layer (GCIPL), inner nuclear layer to the inner boundary of the retinal pigment epithelium (INL-RPE), which contains the photoreceptor layers, and retinal pigment epithelium (RPE). We chose these retinal layers because their age-related changes have previously been researched using the UK Biobank database.^12^, ^13^, ^14^ We report the average thickness as well as the effect of age and sex for each of the 4 layers. We further calculated these measures in each of the following 5 subfields of the retina, the outer temporal subfield, inner temporal subfield, central subfield, inner nasal subfield, and outer nasal subfield. Because the analysis was conducted using 2-dimensional images, we cannot report results for the superior and inferior subfields. To better compare our findings with those of other studies, we corrected our 1-dimensional measurements by assuming the same thickness profile in the entire 2-dimensional subfield.

## Results

### Counterfactual OCT Images to Visualize the Impact of Healthy Retinal Aging and Subject Sex

**Figure 3:**
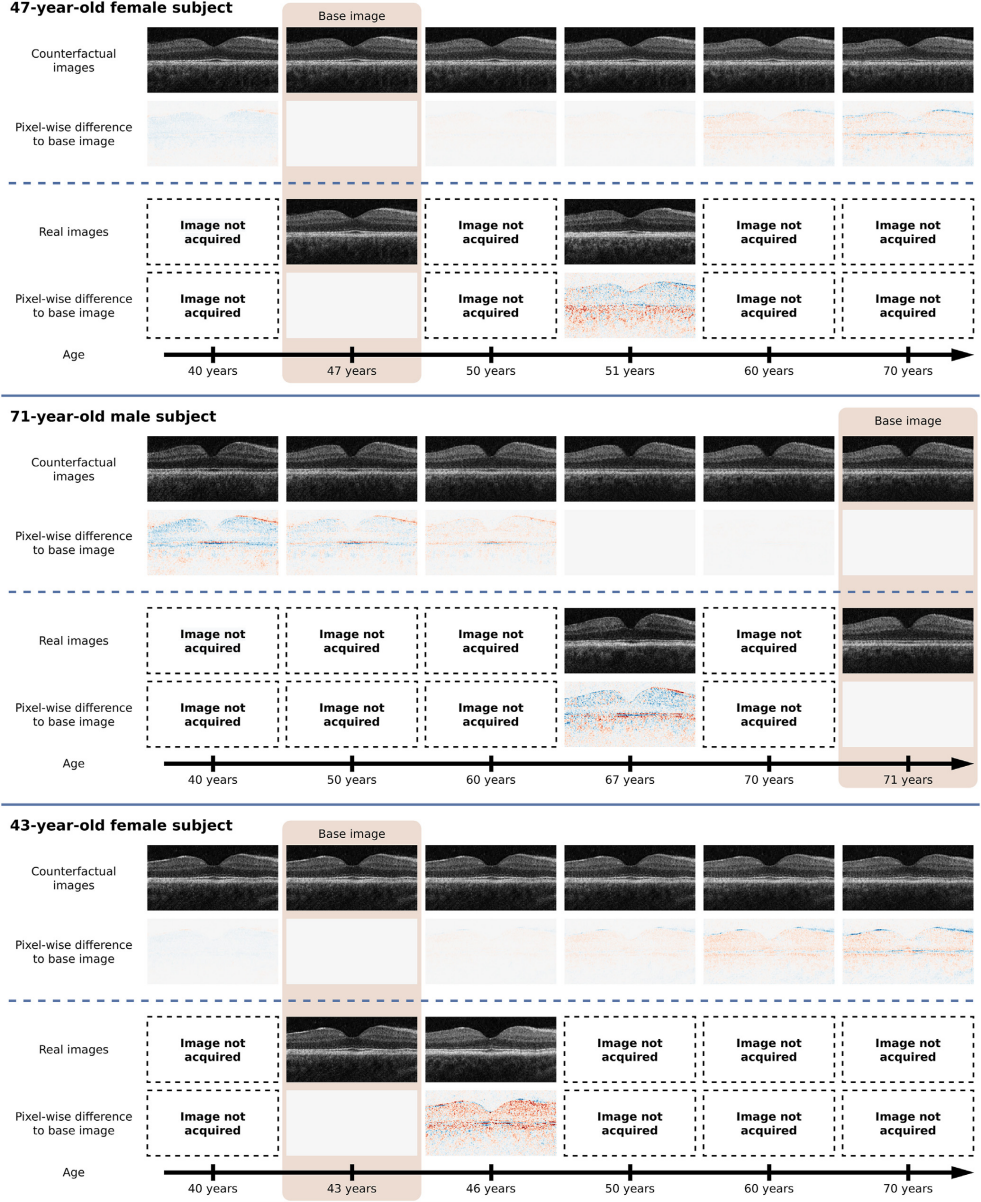
The counterfactual generative adversarial network smoothly visualizes the process of healthy retinal aging at a subject-specific level. In each of the 3 representative examples, the first row presents the counterfactual time series as a function of age. The third row shows the 2 available real images from the UK Biobank data set. The second and fourth rows depict the pixel-wise difference between the time series image and the base image. Red and blue color denote image regions in which the counterfactual is brighter and darker, respectively.

Our counterfactual GAN can smoothly visualize the individual course of retinal aging. Based on a single input image, it provides a plausible hypothesis of how a specific eye will look several decades into the future or how it appeared in the past (Fig 3). By comparison, population-based approaches are limited with regard to the availability, frequency, and range of time series data. In the case of the UK Biobank data set, follow-up OCT scans were acquired from < 5% of the subjects and were dated only 2 to 4 years after the initial scan. Retinal layer orientation, image brightness, and contrast are preserved in the counterfactual images, whereas it fluctuates in the follow-up scans of the UK Biobank data set. This allows focusing on subtle retinal changes, which are difficult to appreciate in conventionally acquired time series. When visually inspecting the generated counterfactual time series, we found that increased age was associated with changes in several retinal layers, including the RNFL, photoreceptor layers, and RPE. This agrees with the previously reported findings.^5^^,^^6^^,^^8^^,^^10^, ^11^, ^12^, ^13^, ^14^

**Figure 4:**
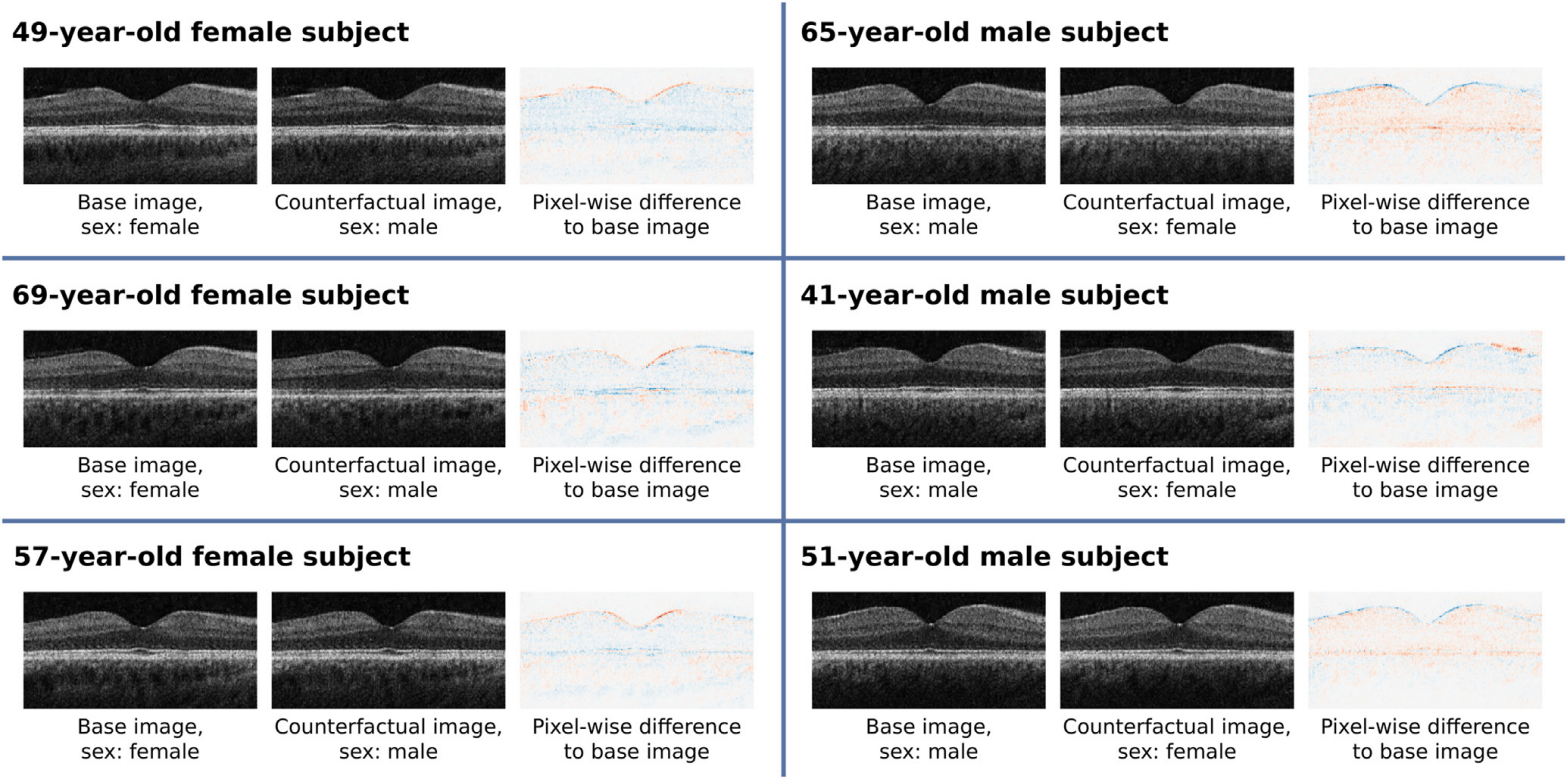
Six representative examples in which the counterfactual generative adversarial network alters a real retinal OCT scan (left image) to appear as if the subject was born as the opposite sex (middle image). The pixel-wise difference between the 2 images is also shown (right image). In red areas the counterfactual is brighter than the base image and in blue regions the counterfactual is darker.

The counterfactual GAN can also simulate how an individual’s eye would appear if the subject had been born as the opposite sex (Fig 4). Naturally, this counterfactual scenario is not possible to research in population-based studies. When changing female eyes to a male appearance, we consistently observe that the foveal pit becomes slightly deeper and steeper. In many cases, the overall macular thickness increases. Conversely, when counterfactually converting male eyes to female, the retina becomes shallower and thinner.

### Benchmarking of Counterfactual OCT Images

**Figure 5:**
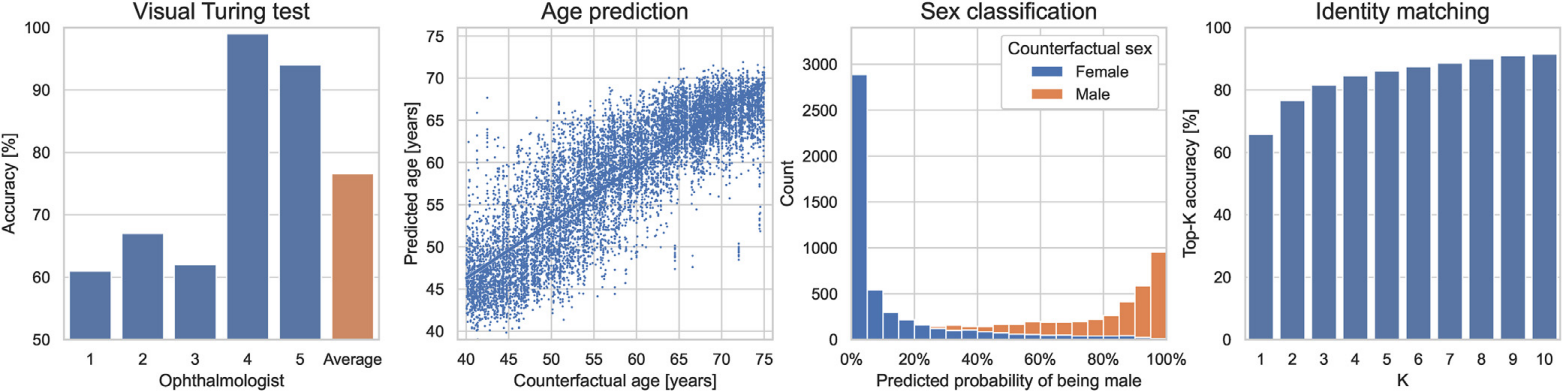
Benchmarking of the counterfactual OCT images. The visual Turing test assessed the images’ realism (left-most graph). Referee neural networks determined the subject’s age and sex from the counterfactual images. We measured whether their prediction agreed with the counterfactual query (middle 2 graphs). A third referee neural network matched counterfactual right eyes with real left eyes. We assessed whether the correct pairing is among the top K guesses of the network as this indicated that the subject identity was preserved in the counterfactual images (right-most graph).

The realism of the counterfactual OCT images was quantified in a visual Turing test. The mean accuracy across the 5 ophthalmologists was 76.6% ± 18.4% (Fig 5). Although this is substantially better than random choice (i.e., 50% accuracy), the ophthalmologists were not able to correctly distinguish whether an image was real or artificially generated in many cases. Two ophthalmologists achieved a substantially higher accuracy of 98% and 94%, respectively, by looking at the choroid and vitreous in the background of the images. They also relied on spotting pathologic features as well as shadowing artifacts caused by blood vessels because these would mostly occur in real OCT images. All ophthalmologists agreed that the counterfactual GAN produces samples with a highly realistic-looking retinal layer structure, which we focus on in the remaining study.

Next, we used the referee networks to predict age, sex, and identity from counterfactual images. We measured whether these attributes matched the corresponding counterfactual queries. The age estimated by the age prediction network agreed with the counterfactual query with a mean absolute error of 4.2 years ± 0.4 years, while being strongly correlated (Pearson’s *R* of 0.86 ± 0.02; Fig 5). The sex classification network correctly predicted the counterfactual sex with an accuracy of 79.7% ± 5.8% and area under the receiver operating characteristic curve of 0.92 ± 0.03 (Fig 5). Finally, we tested whether the identity was preserved in the counterfactual OCT images. We counterfactually increased the age in 528 OCT scans to match the subject’s age at the time of a follow-up scan. The identity-matching network compared the images of the artificially aged right eyes with images of the real left eyes. In 65.8% ± 9.1% of the cases, the referee network correctly matched the right eye to its corresponding left eye, while being given 1000 candidate eyes (top-1 accuracy; Fig 5). In 91.5% ± 3.3% of cases, the correct eye is among the top 10 guesses (top-10 accuracy). Even considering the residual error of all referee networks, these results quantitatively confirm that the counterfactual GAN is able to faithfully simulate the effect of age and sex on the retina, while preserving the identity of the subject.

### Retinal Layer Structure in Counterfactual Images

**Figure 6:**
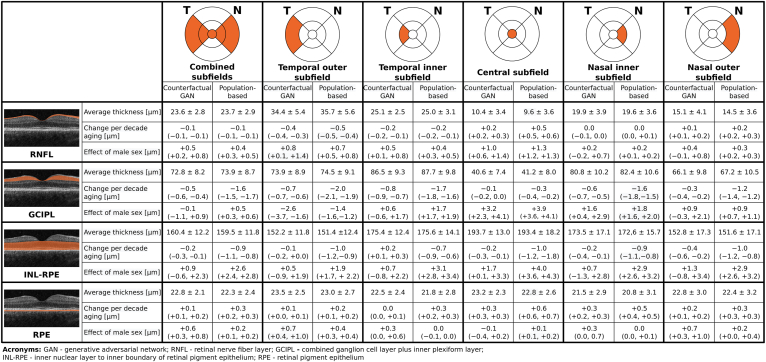
Average thickness, change per decade aging, and effect of male sex for 4 different retinal layer structures and 5 retinal subfields. In each case, we compare the findings obtained by analyzing the counterfactual images (counterfactual GAN) and real OCT images (population-based). The average thickness is reported as mean with its standard deviation, whereas the changes caused by aging and sex are listed as average difference and their 95% confidence intervals.

We segmented and analyzed the RNFL, GCIPL, INL-RPE, and RPE in the counterfactual images. Figure 6 presents their mean thickness as well as the change per decade aging and effect of subject sex. The thickness of the RNFL, GCIPL, and INL-RPE decreases as we increase the counterfactual age, whereas the RPE grows slightly with age. The RNFL and RPE are thicker in male subjects than in female subjects. We also obtained the same set of measurements directly from the real OCT images that were used to train the counterfactual GAN. This approach is similar to a conventional population-based study. The absolute thickness of the 4 retinal structures is very similar in the 2 different approaches. The counterfactual GAN accurately learned to model the impact of age and sex in the RNFL and RPE, while slightly underestimating the effect in the large GCIPL and INL-RPE structures.

## Discussion

In this study, we have created a counterfactual GAN to investigate the individual course of retinal changes caused by healthy aging. Learning from a data set with only 1 OCT image per subject, our machine learning algorithm is able to generate synthetic longitudinal time series. It can visualize how the retina may develop with age at a subject-specific level. This allows studying of subtle structural changes that occur over the course of decades and cannot be resolved in conventional population-based studies. The counterfactual GAN can also simulate how a given eye would look if a person was born as the opposite sex, a scenario that cannot be researched naturally. In extensive benchmarking experiments, we confirmed that our tool creates realistic OCT images and faithfully models the influence of subject age, sex, and identity on the retina.

Our results agree well with previous studies based on the same cohort from the UK Biobank population study.^12^, ^13^, ^14^ Khawaja et al^14^ reported very similar absolute thicknesses and impact of aging for the RNFL and GCIPL. They found that both structures are thinner in male than in female subjects, whereas we found that sex only has a small impact on the GCIPL and the opposite effect on the RNFL. The association between age and photoreceptor thickness was researched by Chua et al.^13^ Our measured INL-RPE thickness was slightly larger than the reported value. The observed relationship between thickness and age was the same in their study and ours. Ko et al^12^ measured the RPE-Bruch membrane complex. In both their study and ours, the RPE thickness was barely affected by subject sex. They found that the complex thins with increasing age in subjects that are ≥ 45 years old, which we did not observe. These differences are potentially caused by our layer segmentation algorithm not fully outlining the fine Bruch membrane in the lower-resolution images. This hypothesis is supported by the fact that we detect a slightly thinner RPE structure compared with their reported thicknesses. In all 3 comparisons, minor discrepancies may also result from the use of 2-dimensional instead of 3-dimensional images in our study and different algorithms for retinal layer segmentation.

Previous work has explored the use of GANs for a range of tasks in the field of ophthalmology, such as image denoising, superresolution, and domain transfer.^27^, ^28^, ^29^, ^30^ In these applications, GANs alter medical images to reflect an improved or functionally different image acquisition process. Ideally, any image transformations would not change information content related to the patient. Conversely, our study researches the setting in which images are altered to reflect changes in the imaged subjects themselves, whereas the acquisition settings are kept fixed. To our knowledge, there is only 1 other study exploring counterfactual image generation for biomarker discovery in ophthalmology. Narayanaswamy et al^31^ have previously proposed a counterfactual synthesis of color fundus photographs to discover indicators of diabetic macular edema. They found that the disease state is linked to the presence of exudates, a known biomarker for diabetic macular edema, as well as a darkening of the foveal region, which is currently not being used for clinical predictions. However, they have not quantitatively assessed the quality of the counterfactual images and did not extract imaging biomarkers from the images. Nonetheless, their study showcases an exciting usage for our tool, modeling the effect of ocular disease on the eye.

At the moment, our counterfactual GAN assumes that the eye’s appearance in OCT images is governed independently by the subject’s age, sex, and identity. Although we aimed to exclude any patients affected by serious eye disease, some eyes with early-stage disease potentially remain in the training data set. The GAN may inadvertently learn to correlate these disease features with age or sex and alter them when generating counterfactual images. To avoid such artifacts, future work could see the creation of a more sophisticated causal model and its integration with a GAN.^32^^,^^33^ Such a model could include and explicitly model the relationship between the eye’s appearance and subject genotype, lifestyle, or retinal diseases. However, this requires the availability of corresponding labels in the data set that is used to train the GAN. Furthermore, the algorithm cannot learn the relationship for groups of subjects that it has not seen in the data set. For example, our GAN has been trained on subjects between the ages of 40 and 75 years. It is not able to model how the eye develops in children, young adults, or individuals that are > 75 years old. Finally, the counterfactual GAN currently only generates 2-dimensional OCT images. Although generating volumetric images with GANs is challenging,^34^ future work should look to increase in the images’ dimensionality as well as their field-of-view and resolution.

In conclusion, this study has demonstrated how counterfactual GANs can aid research into retinal aging by synthesizing high-resolution, high-fidelity OCT images, and longitudinal time series. Ultimately, we envision that they will enable clinical experts to derive and explore hypotheses for potential imaging biomarkers for healthy and pathologic aging that can be refined and tested in prospective clinical trials.

## Supplementary Data

APPLICATION
