# Supplementary material for: Exploring Healthy Retinal Aging with Deep Learning
Source: Ophthalmol Sci. 2023 Mar 1;3(3):100294. doi: 10.1016/j.xops.2023.100294 (PMC10127123; doi:10.1016/j.xops.2023.100294)
Supplement: APPLICATION [file mmc1.pdf]

Design of the counterfactual generative adversarial network

For the task of generating counterfactual OCT images we adapted the GAN framework for image translation by Choi *et al.*[1] Our counterfactual GAN changes existing images according to a counterfactual query that specifies the target age and sex. Female or male sex are encoded as binary label and age is provided as continuous label, which is normalized using the mean and standard deviation of the training dataset distribution. This vector is channel-wise concatenated with the OCT image as input to the generator.

The generator consists of six blocks: two downsampling blocks, two convolutional blocks and two upsampling blocks. The discriminator consists of five downsampling blocks. Each block contains a batch normalization layer, leaky-ReLU non-linearity (slope $= 0.1$) and convolutional layer (up to 64 channels in the generator and 512 channels in the disciminator, kernel size $= 3$, padding margin $= 1$). Upsampling layers in the generator use nearest neighbor interpolation and are placed after the non-linearity.[2] Downsampling in the generator and discriminator is done via strided convolutions. Additionally, we use spectral normalization in both generator and discriminator.[3,4] During prototyping, we experimented with other techniques that did not end up being used in our final network architecture. Most noteworthy, we were not able to achieve equally good results when adding residual connections between network layers.[5,6] Using adaptive instance normalization, which has been previously shown to enhance natural image generation, also did not improve image quality in our experiments.[7,8]

The loss functions used to train the GAN's generator $\mathcal{L}_G$ and discriminator $\mathcal{L}_D$ are given as follows:

$$\mathcal{L}_G = -\log D(G(x,c)) + \gamma_1 \mathcal{L}_{aux}(G(x,c),c) + \gamma_2 \mathcal{L}_{cyc}(G(G(x,c),y),x), \quad (1)$$
$$\mathcal{L}_D = -\log D(x) - \log(1 - D(G(x,c)) + \gamma_1 \mathcal{L}_{aux}(x,y). \quad (2)$$

Here, $G(\cdot)$ and $D(\cdot)$ stand for the generator and discriminator, respectively. x denotes the training images, y the ground truth labels and c the labels that encode the counterfactual query. In addition to the modified minimax loss,[9] the total loss includes two additional terms, $\mathcal{L}_{aux}$ and $\mathcal{L}_{cyc}$, which are weighted by γ_1 and γ_2, respectively. The auxiliary loss $\mathcal{L}_{aux}$ evaluates the ability of the discriminator to predict the subject age and sex. The discriminator's predictions are compared to the ground truth labels for real images and counterfactual queries for the artificially generated images. Predicted sex is compared to the labels via binary cross entropy. Predicted age of a subject is compared to the labels via the L_2 distance. The cycle consistency loss $\mathcal{L}_{cyc}$ encourages the generator to preserve the eyes' identities by generating images that can be converted back to their original appearances. It is calculated by passing a generated counterfactual image through the generator once again to reflect its original attributes and then calculating the L_1 distance between this twice modified image and the original input image.

The network was trained for 250 epochs with a batch size of 256 using the Adam optimizer (learning rate $= 0.001$, $\beta_1 = 0.5$, $\beta_2 = 0.999$).[10] We updated the weights of the discriminator once for every five updates of the generator. We empirically set γ_1 and γ_2 to 1.0 and 10.0, respectively. Following each epoch, we evaluated the model's performance by calculating the Frechét inception distance between the generated samples and our validation dataset.[11] The model instance with the lowest Frechét inception distance was selected for the final evaluation.

Architecture and training of the referee networks

In order to quantitatively assess the age, sex and identity of generated counterfactual images, we trained three Resnet50 convolutional neural networks.[5] For network development and training we used a dataset partition that was not used during training, validation or testing of the generative networks. This dataset was split yet again into training, validation and testing dataset with a ratio of 80% to 10% to 10%.

Determining the subject's age from their OCT image was framed as a regression problem using the L_2 distance as loss function. Sex prediction was treated as a classification task with binary cross entropy as loss. These two networks were trained with the Adam optimizer (learning rate $= 0.001$, $\beta_1 = 0.5$, $\beta_2 = 0.999$) and a batch size of 64 for 250 epochs. We used random scaling, shifts and added gaussian noise as augmentation during training. The network iteration scoring highest on the validation dataset was selected as the final network.

In order to determine whether the subjects identity was preserved, we used a Resnet50 that outputs a 128-dimensional embedding based on the eye's identity. This network was incentivized to minimize the L_2 distance between the embeddings of a left and a right eye belonging to the same subject and maximize the distance when the eyes have different identities. The network was trained using a triplet loss as it has been shown to perform competitively with more complex metric learning approaches.[12] In each batch of 64 eye pairs, only the hardest negative case was considered when calculating the loss as suggested by.[13] The network was optimized using the Adam optimizer for 250 epochs, after which we selected the best performing network iteration.

Architecture and training of the retinal layer segmentation networks

To segment the retinal layers in real and artificial OCT images, we adapt the approach by Shah *et al.*[14] Their method trains a neural network to predict the column-wise coordinates of the surfaces of all retinal layers (i.e. the positions of the surfaces in each OCT A-scan). We use the same dataset as described in the previous section to train a Resnet-50 network that outputs the positions of 2464 coordinates at once (the position of 11 retinal layer surfaces in 224 image columns). We use the L_2 distance between the predicted and ground truth surface coordinates as loss and optimize the network weights for 250 epochs using the Adam optimizer (learning rate $= 0.001$, $\beta_1 = 0.5$, $\beta_2 = 0.999$). We do not use any data augmentation during training.

References

[1] Choi Y, Choi M, Kim M, Ha JW, Kim S, Choo J. Stargan: Unified generative adversarial networks for multi-domain image-to-image translation in *Proceedings of the IEEE conference on computer vision and pattern recognition*:8789–8797 2018.

[2] Odena A, Dumoulin V, Olah C. Deconvolution and checkerboard artifacts *Distill.* 2016;1:e3.

[3] Miyato T, Kataoka T, Koyama M, Yoshida Y. Spectral normalization for generative adversarial networks *arXiv preprint arXiv:1802.05957.* 2018.

[4] Kurach K, Lučić M, Zhai X, Michalski M, Gelly S. A large-scale study on regularization and normalization in GANs in *International Conference on Machine Learning*:3581–3590PMLR 2019.

[5] He K, Zhang X, Ren S, Sun J. Deep residual learning for image recognition in *Proceedings of the IEEE conference on computer vision and pattern recognition*:770–778 2016.

[6] Brock A, Donahue J, Simonyan K. Large Scale GAN Training for High Fidelity Natural Image Synthesis in *International Conference on Learning Representations* 2018.

[7] Huang X, Belongie S. Arbitrary style transfer in real-time with adaptive instance normalization in *Proceedings of the IEEE International Conference on Computer Vision*:1501–1510 2017.

[8] Kim J, Kim M, Kang H, Lee KH. U-GAT-IT: Unsupervised Generative Attentional Networks with Adaptive Layer-Instance Normalization for Image-to-Image Translation in *International Conference on Learning Representations* 2019.

[9] Goodfellow I, Pouget-Abadie J, Mirza M, et al. Generative adversarial nets *Advances in neural information processing systems.* 2014;27.

[10] Kingma DP, Ba J. Adam: A Method for Stochastic Optimization in *3rd International Conference on Learning Representations, ICLR 2015, San Diego, CA, USA, May 7-9, 2015, Conference Track Proceedings* 2015.

[11] Heusel M, Ramsauer H, Unterthiner T, Nessler B, Hochreiter S. Gans trained by a two time-scale update rule converge to a local nash equilibrium *Advances in neural information processing systems.* 2017;30.

[12] Musgrave K, Belongie S, Lim SN. A metric learning reality check in *European Conference on Computer Vision*:681–699Springer 2020.

[13] Hermans A, Beyer L, Leibe B. In defense of the triplet loss for person re-identification *arXiv preprint arXiv:1703.07737.* 2017.

[14] Shah A, Zhou L, Abrámoff MD, Wu X. Multiple surface segmentation using convolution neural nets: application to retinal layer segmentation in OCT images *Biomedical optics express.* 2018;9:4509–4526.
